# Mealtime Environment and Control of Food Intake in Healthy Children and in Children with Gastrointestinal Diseases

**DOI:** 10.3390/children8020077

**Published:** 2021-01-23

**Authors:** Katerina Sdravou, Elpida Emmanouilidou-Fotoulaki, Athanasia Printza, Elias Andreoulakis, Athanasios Evangeliou, Maria Fotoulaki

**Affiliations:** 1 4th Department of Pediatrics, School of Medicine, Aristotle University of Thessaloniki, General “Papageorgiou” Hospital, 56403 Thessaloniki, Greece; elpisemfot@hotmail.com (E.E.-F.); aeevange@auth.gr (A.E.); mfotoul@otenet.gr (M.F.); 2 1st E.N.T. Department, School of Medicine, Aristotle University of Thessaloniki, 54124 Thessaloniki, Greece; nan@med.auth.gr; 3 Hellenic Centre for Mental Health and Research, Adult Psychiatric Unit, Department of Thessaloniki, 55337 Thessaloniki, Greece; eliasandreoulakis@gmail.com

**Keywords:** feeding problems, mealtime structure, food intake, parental feeding practices, gastrointestinal diseases

## Abstract

Parental feeding practices and mealtime routine significantly influence a child’s eating behavior. The aim of this study was to investigate the mealtime environment in healthy children and children with gastrointestinal diseases. We conducted a cross-sectional case–control study among 787 healthy, typically developing children and 141 children with gastrointestinal diseases, aged two to seven years. Parents were asked to provide data on demographics and describe their mealtime environment by answering to 24 closed-ended questions. It was found that the majority of the children had the same number of meals every day and at the same hour. Parents of both groups exerted considerable control on the child’s food intake by deciding both when and what their child eats. Almost one third of the parents also decided how much their child eats. The two groups differed significantly in nine of the 24 questions. The study showed that both groups provided structured and consistent mealtime environments. However, a significant proportion of children did not control how much they eat which might impede their ability to self-regulate eating. The presence of a gastrointestinal disease was found to be associated with reduced child autonomy, hampered hunger cues and frequent use of distractions during meals.

## 1. Introduction

Feeding problems are common in healthy infants and toddlers. Approximately 25–45% of typically developing children are reported to experience some type of feeding difficulties [1,2]. For a long time, it has been known that medical disorders (i.e., prematurity, developmental disabilities) with associated gastrointestinal symptoms may lead to feeding problems in early childhood [3,4,5,6]. A growing body of literature has documented that gastrointestinal diseases such as gastroesophageal reflux, eosinophilic esophagitis or food gastrointestinal allergies are the primary organic causes of feeding problems in children with typical development [7,8,9,10,11]. The clinical manifestations of vomiting, constipation, abdominal pain and rectal bleeding and the repeated negative experiences with feeding have been associated with maladaptive feeding behaviors in these pediatric populations [7,11]. Treatment models for childhood feeding problems consecrate on the mealtime environment and the caregivers’ feeding strategies [12,13,14,15,16]. This is mainly because behavioral and environmental components are present in most cases, even when underlying medical conditions are also present [17,18]. Especially, toddlers and young children have a higher frequency of behavioral problems than older children, suggesting that their caregivers may necessitate greater support with addressing feeding behaviors [12,19].

Previous research suggests that normal feeding development and self-regulation of energy intake in early childhood can be facilitated by proving the child with a structured and consistent mealtime environment in which the parents have the responsibility of what, when and where their child eats, whilst the children themselves have control over how much they eat. To this end, great attention should be paid to children’s internal cues of hunger and satiety [20,21,22]. These elements are of paramount importance for children with gastrointestinal diseases who not only face an increased probability of having behavioral feeding problems but also are confronted with another challenge: they are asked to adhere to a challenging treatment, including dietary restrictions and/or specific diets, which may significantly affect child and parent feeding behavior [23,24]. As children often face difficulty adhering to the dietary changes, adaptation to the treatment regimen requires more active involvement of parents during the feeding process. According to a study, children with eosinophilic esophagitis on food allergen restriction diets had significantly less problematic feeding behaviors than those on regular diets [9]. On the other hand, a more extensive exclusion diet has been associated with more feeding problems [11]. Hence, a supportive feeding environment and parental control over eating are essential for toddlers with gastrointestinal diseases to assure better health and feeding behavior outcomes.

Parental control over eating is another factor that has received much attention in the past decade. Notably, existing work has mainly focused on specific measures of parental control such as the pressure to eat and the restriction of unhealthy foods and has yielded contradictory findings, associating these parental strategies with both negative and positive outcomes concerning weight and diet variables [25]. On the other hand, few studies to date have focused on the preservation of a child’s autonomy and control over eating, especially in families that have to adapt to dietary restrictions [15,26,27,28]. However, these are integral components of normal feeding behavior and are increasingly recognized as essential factors for the prevention and treatment of feeding problems.

On the contrary, socially uncoordinated mealtimes, poor parental modeling, inappropriate parental feeding practices and chaotic environments are some of the most substantial risk factors for development and maintenance of feeding issues in early childhood. Children with feeding problems are less likely to have family meals or eat the same food with the rest of their family and more likely to eat away from the table and the dining room [15,29]. Concerning exclusion diets, the similarity of food consumed by the parents and the children with a gastrointestinal disease is an important issue. However, little is known about the adjustment of the eating habits of the whole family and the incorporation of the dietary restrictions in the family context.

The feeding problem literature demonstrates that parents often use distractions (e.g., use of screens, toys and books) to coax their children to come to the table for eating in the first place, to eat beyond their feeling of hunger or satiety or to eat foods children themselves do not prefer [30,31]. Distraction, therefore, might also be used purposefully on behalf of the parents while aiming to exert some control over the child’s eating. Besides feeding difficulties, eating whilst watching TV is associated with higher child Body Mass Index (BMI) and overweight risk [32].

It is well-documented that feeding environment significantly influences children’s feeding behavior, especially during the first years of life [12,13,17,25,33,34]. Mealtime environment is influenced by a number of parent characteristics such as parental age, education and child characteristics such as gender, child age, weight status, birth order and feeding behavior [26,35,36,37,38]. Parents often use a variety of strategies and adjust the feeding environment in response to problematic feeding behaviors [33,39,40]. Some of these practices can be particularly ineffective and reinforce negative child behaviors whereas others may have a positive impact on a child’s feeding [39,40,41]. Structuring strategies are considered to be more effective to promote healthy eating at early childhood but have turned out to be less beneficial for older children [25]. Parental feeding practices are mainly influenced by their perceptions of their children’s weight and feeding behavior [42,43]. For instance, parental perceptions of picky eating have been associated with coercive feeding practices [26]. Respectively, cultural background influences parental perceptions of children’s weight and problematic eating behaviors [43,44,45]. For instance, although frequency of mealtime behaviors in Greek healthy children was similar to Australian and Canadian normative samples, interestingly enough, Greek parents perceived nearly double the amount of feeding behaviors as problematic compared to the other two populations [46]. 

In this context, the present study aims to assess the mealtime environments of Greek children and to investigate associations between the mealtime environment and parental and child demographic characteristics as well as the children’s BMI. In addition, expanding on previous research that has focused primarily on coercive feeding practices in children with feeding problems, this study aims to explore less studied aspects of mealtime environments such as family mealtime routine, mealtime structure and child’s autonomy in children with gastrointestinal diseases.

## 2. Materials and Methods

This cross-sectional case control study was approved by the Institutional Ethics Committee and the Greek Ministry of Education. All parents provided written informed consent before participating in the study.

### 2.1. Participants

A total of 2478 structured sets of questions were distributed to the parents of healthy, typically developing children (control group) and children with gastrointestinal diseases such as gastroesophageal reflux disease, food allergy, enterocolitis, enteropathy and celiac disease (clinical group), aged 2–7 years, and 1181 were returned (response rate: 47.6%). Of them, 928 questionnaires were fully answered by the parents (787 in the control group and 141 in the clinical one). 

The participants of the normative sample were recruited from 75 kindergartens from various geographic regions of Greece. The kindergartens were selected via convenient sample strategy based on representativeness of a large geographical part of Greece, including both rural and urban areas. The primary investigator contacted the headmasters of the kindergartens by phone and asked their permission to participate in the study. In the beginning, the investigator contacted 121 kindergartens. Of them, 75 agreed to take part and 46 denied to participate in the study. The headmasters made preliminary contact with potential participants and distributed the information letter and the set of questions within their schools. Parents wishing to participate filled out the set of questions in a hard copy form, using the paper-and-pencil manner. They did not receive a reminder to participate. All enrolled participants in the clinical group had a confirmed diagnosis of a gastrointestinal disease made by a pediatric gastroenterologist and were patients of a Pediatric Gastroenterology Outpatient Clinic. During their visits, parents were routinely asked to complete the set of questions. It was explicitly stated to the parents that the use of the data for research purposes was not a prerequisite for access to the service. Parents also had the option to take the set of questions home and complete it or discard it privately. The recruitment period lasted from November 2016 to June 2018. Regarding the healthy group, we enrolled a group much larger than needed for the current study sample, as we are simultaneously creating a database of normative data to be available as a reference healthy population for future studies.

Children with medical history of premature birth or any condition that might affect their motor pattern of swallowing such as developmental disorders, craniofacial abnormalities, neurological disorders and other organic diseases were excluded from the study. 

### 2.2. Measures

#### 2.2.1. Demographic and Anthropometric Data

Demographic data of the parents such as sex, age, educational level and employment status alongside demographic data of the children such as sex, age, presence of siblings, birth order and anthropometric data such as birth weight and current height and weight were recorded. Regarding the clinical group, the diagnosis was made by a pediatric gastroenterologist. 

Height and weight were measured at enrollment using a standing stadiometer and hospital scale in the Pediatric Gastroenterology Outpatient Clinic. As for the healthy participants, height and weight were provided by self-report. For both groups, height and weight scores were converted to age- and gender-specific BMI z scores using the World Health Organization’s Anthro and AnthroPlus software by the principal investigator.

#### 2.2.2. Mealtime Environment

Data were collected as part of a larger project comparing predictors of pediatric feeding difficulties in healthy children and children with gastrointestinal diseases. For the purposes of the larger project, we used three measures including one validated questionnaire to assess feeding behavior and two sets of questions to address parental feeding practices in response to feeding problems and mealtime environment, respectively. The aspects of mealtime environment that were to be assessed in this study were determined by a multidisciplinary group of experts on feeding and swallowing problems, consisting of two speech and language therapists, a pediatric gastroenterologist and an otolaryngologist. Inclusion of items was guided by the intent to reflect three broad dimensions of healthy mealtime environment: mealtime structure that creates a supportive environment of healthy eating, parental feeding practices that influence a child’s self-regulation of intake and a child’s behaviors that indicate ability to self-regulate intake.

The existent validated measures on child and parental feeding behavior include items that address several aspects of child and parental feeding behavior and do not only focus on structure or control of intake. For this reason, we decided to conclude to a set of questions referring exclusively to certain aspects of mealtime environment. Hence, we have excluded questions about children’s problematic feeding behaviors (such as food refusal, selectivity or oral motor problems), as well as parental strategies and feelings regarding children’s feeding, since all of these were already assessed by the two measures previously mentioned. In addition, we did not include parental control practices for promoting well-balanced food intake and weight control (monitoring healthy food choices, covert restriction and overt restriction) or regulating the child’s emotional states (e.g., reward for behavior), since our initial objective was to examine factors associated with feeding problems and not obesity. Finally, no questions regarding the child’s body image perception (e.g., “my child says she/he’ll get fat if she/he eats too much”) were included, for these are behaviors that older children exhibit and are connected mainly to eating disorders.

Initially, a review of the literature and measures was conducted to identify an extensive list of items reflecting the following dimensions of mealtime routine: mealtime structure, mealtime schedule, child control of intake, parent control of intake and the communication between a child and a parent regarding the child’s feelings of hunger or satiety. Relevant items from the Children’s Eating Behavior Inventory (CEBI) [47], the Feeding Practices and Structure Questionnaire (FPSQ) [20], the Feeding Strategies Questionnaire (FSQ) [48] and the Comprehensive Feeding Practices Questionnaire (CFPQ) [49] were identified. Furthermore, the 4 researchers participating in the process suggested, each one separately, certain questions that they considered relevant to this topic, based on the feeding problem literature and interviews they had with parents during their clinical practice. Afterwards, a discussion was held among the researchers to eliminate similar items or to exclude items that had not been approved by everyone. Finally, they reached a consensus for the 24 items included in this set of questions. A pilot interview of ten Greek parents of healthy children and children with gastrointestinal diseases aged 2 to 7 years was conducted to confirm the clarity and appropriateness of the questions.

A comprehensive set of 24 Likert-type questions was finally used to investigate certain aspects of mealtime environment, parental practices and children’s behavior such as the mealtime routine of the family (3 items), the mealtime structure (10 items), the child’s control of intake (3 items), the parental control of intake (4 items) and the communication between the child and parent regarding the child’s feelings of hunger or satiety (4 items). Each item was measured on a five-point Likert-type scale ranging from 1 = “never” to 5 = “always or almost always” (Appendix A). 

## 3. Statistical Analysis

In the descriptive part of the statistical analysis, absolute (number) and relative (%) frequencies were calculated for the categorical variables (demographic data) and means and standard deviation were calculated for the scale (anthropometric data) or ordinal variables (the answers to the 24 questions). In the analytical part, chi-square and Mann–Whitney U tests were performed to compare the two groups as appropriate, depending on the type of variables. The association between demographic or anthropometric characteristics and the frequency of parental feeding practices was examined by calculating chi-square and Spearman’s nonparametric correlation coefficients, accordingly. The level of statistical significance (alpha) was set at *p* < 0.05. The a priori sample size estimation was carried out using GPower v3.1 software [50]. SPSS Statistics v20 statistical software (IBM, 142 Armonk, New York, NY, USA) was used for the analysis. 

## 4. Results

### 4.1. Mealtime Environment in Healthy Children and in Children with Gastrointestinal Diseases

The demographic and anthropometric characteristics of the two groups are presented in Table 1. Diagnoses of the clinical group are presented in Table 2.

The mean values of the answers to each of the 24 questions in the two subgroups alongside with a between-groups comparison are presented in Table 3. Regarding the mean scores of the answers to the 24 items, the two groups differed significantly in nine of the 24 questions, namely questions 1, 4, 5, 6, 9, 10, 11, 12 and 16. A similar comparison was performed by means of the chi-square test (Table S0). In this case, it was not the means but the frequencies of the answers that were compared. Of note, in order to make the inferences more easily drawn, the five-point answers were also coded as two-point, with answering “1. Never”, “2. Rarely” and “3. Sometimes” being coded as “Relatively rare” and answering “4. Often” and “5. Very often or Always” coded as “Relatively often”. In this type of comparison, significant differences were found in questions 4, 5, 6, 8, 9, 10, 11, 12, 16 and 22 when the five-point answers were compared (very similar to the case of means comparison mentioned above, except for question 1, question 8 and question 22) and in questions 1, 6, 9, 10, 11, 12 and 16 when the two-point answers were compared. 

Both groups provided quite structured mealtimes and consistent mealtime schedules. The majority of the children have their meals at the same hour every day (95.3% of healthy children and 94.3% of clinical group) and have the same number of meals every day (86.1% of healthy group and 87.9% of clinical group). Likewise, most parents provide opportunities of modeling by eating along with their children (83% of healthy children and 76.6% of clinical group). Parents of both groups exhibit high control of their child’s food intake by deciding both when it is time for their child to eat (76.1% of healthy children and 70% of clinical group) and what kind of food their child should eat (84.8% of healthy children and 81.9% of clinical group). Almost one third of the parents also decide how much their child eats (35.7% of healthy children and 37.6% of clinical group).

In contrast with the healthy children, the children with gastrointestinal diseases were found to be less autonomous during feeding (question 9—Q9) (*p* < 0.001), less likely to communicate hunger (Q17) (*p* < 0.01), less likely to eat the same food as their family (Q6) (*p* < 0.001) or sit at the table during meals (Q10) (*p* < 0.001). Instead, they were more likely to watch TV (Q11) (*p* < 0.01) or take along toys during meals (Q12) (*p* < 0.001). 

### 4.2. Influence of Demographic Characteristics on Mealtime Environment

As a next step, we went on to investigate (in each sample, separately) whether any of the demographic variables examined were associated with the frequency of the mealtime behaviors assessed. For this purpose, we used the condensed two-point coding that readily provided inferences and differentiation between frequent and infrequent behaviors. 

In the control group, the results were as follows. 

Girls were more likely to help with meal preparation (Q4), to eat autonomously (Q9), to sit at the table during meal (Q10) and to express satiety (Q17). Boys were more likely to eat the same number of meals every day (Q8) and to be encouraged to clean their plate (Q23). Single children were more likely to eat depending on their appetite (Q24) but less likely to eat with the family (Q5), to eat the same food as the family (Q6), to express hunger (Q16), to sit at the table (Q10) and to eat autonomously (Q9). Firstborns were more likely to bring toys (Q12) and watch TV during meals (Q11) and less likely to eat autonomously (Q9), sit at the table (Q10) and express hunger (Q16). Children aged 5 years or younger were more likely to have control over how much they eat (Q13) and less likely to eat autonomously (Q9) and choose their snacks (Q21). Fathers were less likely to decide when their child eats (Q18) and to be notified about their child’s hunger (Q16). Sex concordance (the parent and the child being of the same sex) was associated with a higher likelihood of expressing satiety (Q17) and lower likelihood of helping with meal preparation (Q4), watching TV (Q11) (on behalf of the child) and urging (on behalf of the parent) to “clean the plate” (Q23). Parental age group (below or above 40 years) was not associated with any of the 24 questions. Parents with relatively lower education (12 years or less) were more likely to provide opportunities of modeling by eating at the table (Q1), having family meals (Q2) and eating with the child (Q5). Their children, however, were less likely to have a standard time (Q7) or number of daily meals (Q8). Unemployed parents were less likely to encourage their child to help with meal preparation (Q4) and more likely to be notified by their child about satiety. The aforementioned results are presented in Tables S1, S2 and S3.

A similar analysis in the clinical group yielded the following results: 

Girls were more likely to help with meal preparation (Q4) and less likely to bring toys to the table (Q12). Single children were more likely to have control over how much they eat (Q13) and to be allowed to sit up from the table during the meal (Q22). Being the firstborn child was not associated with any of the 24 answers. Children aged 5 years old or younger were less likely to eat with the family (Q5), eat the same food as family (Q6), eat autonomously (Q9) and sit at the table during meal (Q10). Fathers more rarely let their child decide what he/she is going to eat (Q15). Sex concordance (the parent and the child being of the same sex) was associated with a higher likelihood of helping at meal preparation (Q4) and a lower likelihood of bringing toys at meals (Q12) on behalf of the child. The parental age group (below or above 40 years) and the educational level of the parent were not associated with any of the 24 questions. Unemployed parents were more likely to provide daily family meals (Q2). The aforementioned results are presented in Tables S4–S6. A comparison between the two groups (by means of a two-layer chi-square test) is presented in Table S7.

### 4.3. Association of zBMI and Birth Weight with Mealtime Environment

In the control group, zBMI was positively associated with autonomy (Q9), notification about hunger (Q16), parental respect of the child’s appetite (Q24) and negatively associated with distraction, bringing toys along (Q12) and parental urging to eat (Q23). Birth weight was associated only with autonomy (Q9). However, the strength of the aforementioned correlations was rather weak. 

In the clinical group, zBMI was positively associated with eating the same food as the family (Q6), autonomy (Q9) and sitting at the table (Q10) and negatively associated with family meals (Q2). Birth weight was associated with eating the same food as the family (Q6), autonomy (Q9), child’s decisiveness on amount of food (Q13) and notification concerning hunger (Q16). No negative association was found. The results are presented in Table 4.

## 5. Discussion

The present study showed that feeding environment was structured and consistent (Q4–12 and Q22) and that a family mealtime routine (Q1–3) was provided to the majority of children in both groups. This is a favorable condition for the population of the study considering that recent research suggests that better dietary outcomes are expected when structure-related practices are followed [51,52]. However, children with gastrointestinal disorders were found to sit at the table during the meal or eat the same food as the rest of the family less often compared to the children of the control group. This finding is in line with several studies that have demonstrated that children with feeding disorders are more frequently fed away from the family table [29,30]. Eating separately might be indicative of a feeding problem and should probably be a red flag for the whole family. In addition, the tendency of children in the clinical group to eat the same food as the rest of the family less often has already been associated with feeding problems in previous studies [15,29].

Despite the quite structured meal environment, distractions during the meal (watching TV or bringing toys along) were found to be rather frequent in both groups (30.9% of the healthy children and 41.8% of the children with a gastrointestinal disease), particularly in the clinical one. Considerable evidence has highlighted distraction during feeding as a predictive factor of feeding problems [31]. Moreover, recent studies have reported that watching TV while eating results in lower-quality dietary habits characterized by a higher energy intake and, consequently, higher BMI in preschool children [53]. 

Another point of differentiation between the two groups was that children with gastrointestinal diseases were found to be less autonomous during feeding (Q9) since they needed an adult’s help at a significantly higher percentage (38.3% vs. 14.55% of the children in the healthy group). Autonomous feeding is an important developmental stage that can be partially achieved by the age of 12 months. Failure to develop self-feeding has been associated with the presence of feeding problems [12] as well as with non-IgE (Immunoglobulin E)-mediated allergic gastrointestinal disorders [54]. Moreover, recent studies support that baby-led weaning promotes self-feeding during the introduction of solid foods, has a positive effect on self-regulation of appetite in children aged 18–24 months [55] and is also associated with lower selectivity rates [56].

The above findings might be partially attributed to specific or exclusion diets that children with gastrointestinal disease possibly follow. Diet plays a significant role in the management of gastrointestinal diseases, and sometimes, it serves as an exclusive treatment. Parents are encouraged to adapt to specific dietary guidelines such as an exclusion diet and specific feeding times, influencing different aspects of the food environment. This fact may explain the observed lower percentage of autonomy in children with gastrointestinal disorders and the tendency to eat different foods. The associations found in our study can partially reflect this condition. However, the integration of the child’s diet to the family one, to any possible extent, would be beneficial, facilitating the adaptation of the diet and contributing to the acceptance of new food and adoption of healthier eating habits [15]. Furthermore, autonomy could also reflect whether children have adapted to their special needs, and aiming to improve autonomy may result in better application of these treatments. In some cases, however, the parent’s attitude is shaped to such an extent that it can remain even after the gastrointestinal problem is resolved, leading to secondary influences on feeding environment [57].

Another issue that deserves attention is the communication of satiety and hunger cues (Q16–17). The study showed that although child–parent interaction (notification about hunger) during feeding was relatively common in both groups, children with gastrointestinal diseases were less likely to express hunger in comparison to the typically developing children. The present study has also shown that the parents often disregarded their child’s satiety in that the children in both groups were often urged to “clean their plate” (Q23) regardless of satiety expression. However, to promote optimum feeding, not only should parents be responsible for what and when their children eat, as replicated in the present study (Q18–21), but children should also gain some control over their intake by determining the quantity of their meals [58]. 

Besides the comparison between the two groups in terms of feeding routine and practice, the associations of the latter with certain demographics and the children’s anthropometric characteristics was also investigated within each group separately. The results suggest that the child’s sex, the presence of siblings, the order of birth and parent’s education were the factors that were more profoundly associated with mealtime environment in the group of healthy children, whereas in the clinical group, the child’s age was the factor that differentiated the mealtime practices the most (in four out of the 24 practices that were examined). Taken together, the rough comparison of the two groups and the different pattern of associations between certain demographic factors and the aspects of the mealtime environment that were examined suggest that a gastrointestinal disease per se may be a determining factor of feeding setting and practice that possibly prevails over the influence of demographic factors. The role of demographics was remarkably attenuated compared to their role in the healthy group. Conversely, in healthy children, it appeared that demographic variables being “freed” from the dominance of a certain gastrointestinal disease were able to influence mealtime behaviors to a greater extent. Specifically, the feeding environment of the control group seems less “ideal” for a firstborn, an only child or a boy. An interesting finding is the fact that parents with a low educational level seem to provide more opportunities of modeling (e.g., by eating along with their child and having family meals on a regular basis) while parents with a high educational level place more emphasis on a consistent schedule (same time and same number of meals) and have more control over how much and when the child will eat.

Researchers are increasingly highlighting the significance of environment for the enhancement of feeding development and the reduction in feeding problems. The wide range of mealtime environment aspects that were covered by the 24-item questionnaire (including aspects that are not usually addressed) might contribute to the provision of further insight concerning the pursuit of a favorable mealtime environment. Another important strength is that the study was not limited to the comparison between the two groups but included an investigation for any influence of major demographic characteristics upon the feeding environment. Last but not least, the large size of both samples and particularly that of the healthy children group, which, notably, was collected from a very large geographic range including urban and rural areas, represent some other strengths. 

A limitation of our study is that the response rate in the healthy group, although satisfactory (44.26%), was rather low when compared to that of the clinical group (93%). This might have allowed a selection bias to interfere (in that parents more actively involved in the feeding process or facing some difficulties with feeding might have been more willing to participate) and this might have led to an overestimation of feeding problems in the healthy group. Moreover, there were some differences between the two groups in terms of certain demographics; hence, the presence of a gastrointestinal disorder (i.e., the disease) was not the only between-subjects factor. Therefore, the differences found between the two groups could not be safely attributed solely to the presence or absence of a gastrointestinal disease. That said, a multivariate analysis was beyond the scope and capacity of the present study. In addition, inferences about causality cannot be safely extracted considering that cross-sectional studies do not provide such opportunity. The fact that some of the differences found in the control group were not replicated in the clinical group might also be attributed to the smaller size of the latter, since subtle differences are more difficult to detect in relatively smaller samples. Therefore, wherever, in the present study, a marginally non-significant *p*-value was found within the clinical group, it should be interpreted with caution, as it might have been statistically significant had the sample been as large as the control group. For the healthy participants, height and weight were provided by self-report. However, the parental reports are expected to be rather reliable and near the filling date of the set of questions since, at the age of the participants, height and weight are systematically measured at their schools by trained staff members as well as during their regular visits and appointments with their pediatricians. This study is a part of a larger study that aimed to identify questions that could potentially serve as indicators of a feeding disorder. Therefore, in our case, we considered false negatives far less tolerable than false positives. In other words, falsely identifying a question as a candidate within a screening tool that would later prove to be inappropriate would be more affordable than falsely eliminating a question that might have been useful. The aforementioned rationale alongside the criticism that the correction for multiple comparisons has received led us to decide not to apply any adjustment, although the risk for an inflated type I error is not negligible. Lastly, the questionnaire used for the study had not undergone a formal validation process. Although the information provided by each item is meaningful, its reliability when treated as a single case was found to be rather low (Cronbach’s alpha = 0.552). Acknowledging that the feeding environment is shaped in a cultural context, we wanted to examine every single one of these elements in a large cohort of Greek subjects. Hence, it was not our intention to examine the psychometric properties of an extended tool including all these questions but rather to make it possible to detect the most targeted questions among them that might form a brief questionnaire. Furthermore, culture-specific influences on parental perceptions of a child’s feeding behavior might affect the feeding environment. Therefore, the interpretation of the findings as well as their generalization to other cultures should be tried with caution.

This research provides a comprehensive analysis of feeding environments and encourages similar research in the future in order for targeted and probably family-based prevention programs to be formulated. Parental education might lie at the core of such interventions. Parents need to understand the benefits of providing a structured meal environment, fostering their child’s autonomy and their ability to self-regulate intake. The findings of this study contribute to this goal. To our knowledge, this is the first study to highlight the impact that a gastrointestinal disease exerts on feeding environment. That said, a more thorough investigation in a multivariate context is required in order to recognize and manage the difficulties children with a gastrointestinal disease face during mealtime. Future longitudinal or prospective studies with samples large enough to afford a multivariate analysis that would include factors such as those identified in the present study are needed. 

## 6. Conclusions

In conclusion, the high prevalence of obesity and feeding problems among preschool children remains an issue of major public concern and it is a research priority to identify modifiable parameters in order to ensure effective and efficient interventions early in childhood. Our study results suggest that although parents provide a structured and consistent mealtime environment, a significant proportion of children do not control how much food they eat or how much they watch TV while eating, which might impede their ability to self-regulate eating. Identification and description of diversities and deviation from the ideal mealtime environment, which were found to be more profound in children with gastrointestinal diseases, is a first step towards improvement of potential feeding problems. Improvement of these issues might also contribute to better adaptation of children with gastrointestinal disease to special diets. Moreover, specific demographic factors affect mealtime environment, offering potential fields of early intervention. Our findings provide important new information about the feeding environment which can be used in the development of interventions for feeding problems or obesity. 

## Figures and Tables

**Table 1:** Children’s and parents’ demographic characteristics and children’s anthropometric characteristics in the two groups (healthy children vs. children with gastrointestinal diseases).

		Healthy (*N* = 787) *Ν* (%)	Patients (*N* = 141) *Ν* (%)	Groups Comparison *p*-Value
Child sex	Female Male	394 (50.1%) 393 (49.9%)	64 (45.4%) 77 (54.6%)	0.307
Child age group	≤5 years >5 years	367 (46.6%) 420 (53.4%)	99 (70.2%) 42 (29.8%)	<0.001
Only child	Yes No	183 (23.3%) 604 (76.7%)	46 (32.6%) 95 (67.4%)	0.017
Firstborn	Yes No	411 (52.2%) 376 (47.8%)	91 (64.5%) 50 (35.5%)	0.007
Parental sex	Female Male	730 (92.8%) 57 (7.2%)	136 (96.5%) 5 (3.5%)	0.105
Same sex between child and parent (sex concordance)	Yes No	388 (49.3%) 399 (50.7%)	80 (56.7%) 61 (43.3%)	0.104
Parental age group	<40 years ≥40 years	581 (73.8%) 206 (26.2%)	109 (77.3%) 32 (22.7%)	0.383
Parental education	≤12 years >12 years	362 (46.0%) 425 (54.0%)	49 (34.8%) 92 (65.2%)	0.013
Working parent	Yes No	552 (70.1%) 235 (29.9%)	84 (59.6%) 97 (40.4%)	0.013
Child’s age (years)	Mean ± SD * Median	4.91 ± 1.00 5.17	4.18 ± 1.28 4.42	<0.001
BMI ** (current)	Mean ± SD Median	15.90 ± 2.22 15.61	15.28 ± 1.68 15.02	0.001
BMI z-score (current)	Mean ± SD Median	0.27 ± 1.39 0.23	−0.23 ± 1.31 -0.29	<0.001
Birth weight (grams)	Mean ± SD Median	3263.50 ± 450.10 3220	3212.09 ± 414.54 3160	0.140

Note: Percentages refer to the column, i.e., within each group. The two groups were compared by means of a chi-square or Mann–Whitney U test, accordingly. * SD = Standard Deviation. ** BMI = Body Mass Index.

**Table 2:** Diagnoses in the clinical group. (*N* = 141).

Diagnosis	Frequency (Percentage)
Food protein-induced gastroesophageal reflux disease (GERD)	30 (21.3%)
Food protein-induced enterocolitis syndrome (FPIES)	27 (19.1%)
Gastroesophageal reflux disease (GERD)	14 (9.9%)
Food allergy enteropathy	67 (47.5%)
Celiac disease	3 (2.1%)

**Table 3:** The parents’ answers to each question.

	Healthy Mean ± SD	Patients Mean ± SD	*p* ^a^
**1. We eat at the table**	**4.51 ± 0.84**	**4.28 ± 1.00**	**0.007**
2. We eat at least one meal a day all of us together (or almost all of us)	4.10 ± 1.06	3.99 ± 1.12	0.320
3. We eat at almost the same time every day	4.33 ± 0.90	4.21 ± 1.02	0.220
**4. I encourage my child to help at meal preparation**	**3.49 ± 0.99**	**3.27 ± 1.12**	**0.025**
**5. I or other family members eat with the child**	**4.36 ± 0.85**	**4.13 ± 1.01**	**0.013**
**6. My child eats the same food with the rest of the family**	**4.62 ± 0.77**	**4.29 ± 0.99**	**<0.001**
7. My child eats at almost the same time every day	4.67 ± 0.61	4.63 ± 0.69	0.749
8. My child eats the same amount of meals every day	4.34 ± 0.79	4.30 ± 0.88	0.854
**9. My child eats autonomously (without an adult’s help)**	**4.45 ± 0.84**	**3.79 ± 1.22**	**<0.001**
**10. My child sits at the table during the meal**	**4.58 ± 0.73**	**4.26 ± 1.09**	**0.003**
**11. My child watches TV while eating**	**2.92 ± 1.13**	**3.21 ± 1.19**	**0.007**
**12. My child brings toys (books, tablets, etc.) at the table during the meal**	**2.09 ± 1.15**	**2.39 ± 1.37**	**0.038**
13. My child decides how much he/she is going to eat at every meal	3.83 ± 1.09	3.78 ± 1.25	0.946
14. My child decides when he/she is going to eat every meal	2.63 ± 1.11	2.75 ± 1.17	0.293
15.My child decides what he/she is going to eat from the food I have served	3.26 ± 1.23	3.22 ± 1.29	0.786
**16. My child informs me or conveys to me that he/she is hungry**	**4.43 ± 0.91**	**4.16 ± 1.07**	**0.002**
17.My child informs me or conveys it when he/she is full	4.77 ± 0.55	4.74 ± 0.48	0.231
18. I decide when it is time for my child to eat food	4.07 ± 0.96	3.92 ± 1.09	0.202
19. I decide when it is time for my child to eat a snack	3.77 ± 1.08	3.72 ± 1.07	0.558
20. I decide which food my child is going to eat	4.30 ± 0.92	4.29 ± 0.96	0.900
21.I decide which snacks my child is going to eat	4.07 ± 0.95	4.04 ± 1.07	0.849
22. I allow my child to sit up from the table during the meal if he/she wishes to	2.73 ± 1.06	2.71 ± 1.26	0.437
23. I encourage my child to eat all the food that I put on his/her plate	3.81 ± 1.21	3.99 ± 1.18	0.060
24. I allow my child to eat depending on his/her appetite	4.07 ± 0.96	3.96 ± 1.06	0.325

^a^*p* value for Mann–Whitney U test. *p* values below 0.05 are shown in bold.

**Table 4:** Bivariate correlations between the mean score in Q1–Q24 and birth weight and zBMI *.

	Healthy Children		Children with Gastrointestinal Diseases	
	Birth Weight	zBMI *	Birth Weight	zBMI *
Q1 ^a^	0.020 (0.569)	0.042 (0.259)	−0.006 (0.942)	−0.003 (0.970)
Q2	0.035 (0.327)	0.033 (0.381)	−0.085 (0.319)	−0.180 **(0.040)**
Q3	0.043 (0.232)	0.024 (0.519)	−0.096 (0.319)	−0.152 (0.085)
Q4	0.032 (0.374)	−0.006 (0.880)	0.044 (0.602)	0.098 (0.267)
Q5	0.010 (0.782)	−0.005 (0.901)	0.038 (0.650)	0.025 (0.776)
Q6	0.023 (0.513)	0.033 (0.381)	0.205 **(0.015)**	**0.177 (0.044)**
Q7	0.029 (0.418)	−0.023 (0.541)	0.006 (0.945)	−0.024 (0.790)
Q8	−0.011 (0.755)	−0.055 (0.138)	0.041 (0.630)	−0.082 (0.356)
Q9	0.072 **(0.045**)	0.161 **(<0.001)**	0.231 **(0.006)**	0.280 **(0.001)**
Q10	0.029 (0.409)	0.051 (0.173)	0.132 (0.120)	0.228 **(0.009)**
Q11	−0.035 (0.321)	−0.004 (0.909)	−0.006 (0.944)	0.049 (0.577)
Q12	−0.002 (0.965)	−0.080 **(0.031)**	0.028 (0.744)	−0.100 (0.256)
Q13	0.016 (0.644)	0.048 (0.916)	0.217 **(0.010)**	−0.070 (0.432)
Q14	0.002 (0.957)	−0.010 (0.781)	0.136 (0.108)	0.093 (0.294)
Q15	0.003 (0.938)	0.027 (0.466)	0.075 (0.378)	−0.006 (0.949)
Q16	0.051 (0.153)	0.095 **(0.011)**	0.231 **(0.006)**	0.128 (0.147)
Q17	0.044 (0.221)	0.031 (0.412)	0.114 (0.177)	−0.043 (0.627)
Q18	−0.018 (0.621)	−0.055 (0.142)	0.016 (0.851)	−0.146 (0.098)
Q19	−0.064 (0.075)	−0.035 (0.350)	−0.009 (0.919)	0.056 (0.531)
Q20	−0.004 (0.909)	−0.009 (0.805)	−0.012 (0.887)	−0.008 (0.924)
Q21	−0.038 (0.294)	−0.090 (0.015)	−0.120 (0.160)	−0.026 (0.771)
Q22	−0.002 (0.955)	0.009 (0.817)	0.050 (0.558)	−0.043 (0.631)
Q23	0.025 (0.487)	−0.104 **(0.005)**	−0.049 (0.565)	−0.089 (0.318)
Q24	−0.032 (0.374)	0.075 **(0.045)**	−0.016 (0.850)	−0.044 (0.618)

Notes: ^a^ Q = question. Spearman’s rho correlation coefficients are presented followed by *p*-values in parenthesis; *p*-values lower than 0.05 are shown in bold. * zBMI = standardized body-mass index.

## Data Availability

The data presented in this study are available in the article and the supplementary materials.

## Supplementary Materials

The following are available online at https://www.mdpi.com/2227-9067/8/2/77/s1, Table S0: Frequency distribution of the answers to the scale questions in the two groups. Table S1: The parental practices in association with the child’s sex, birth order and the existence of siblings. Group: healthy children. Table S2: The parental practices in association with the child’s age group, parent’s sex, and child-parent sex concurrence. Group: healthy children. Table S3: The parental practices in association with the parent’s age, education and employment status. Group: healthy children. Table S4: The parental practices in association with the child’s sex, birth order and the existence of siblings. Group: Children with gastrointestinal diseases. Table S5: The parental practices in association with the child’s age group, parent’s sex, and child-parent sex concurrence. Group: Children with gastrointestinal diseases. Table S6: The parental practices in association with the parent’s age, education and employment status. Group: Children with gastrointestinal diseases. Table S7: Associations between items 1–24 and demographics as well with group. Two-layer chi-square.

## Appendix A. The Set of Questions.

**Mealtime Environment and Routine**	**Mealtime Routine May Differ from Family to Family. For Each One of the Questions Following, Please Select the Answer that Best Suits Your Child and Your Family.**				
1. We eat at the table	☐ Never	☐ Rarely	☐ Sometimes	☐ Often	☐ Always
2. We eat at least one meal a day all of us together (or almost all of us)	☐ Never	☐ Rarely	☐ Sometimes	☐ Often	☐ Always
3. We eat at almost the same time every day	☐ Never	☐ Rarely	☐ Sometimes	☐ Often	☐ Always
4. I encourage my child to help at meal preparation	☐ Never	☐ Rarely	☐ Sometimes	☐ Often	☐ Always
5. I or other family members eat with the child	☐ Never	☐ Rarely	☐ Sometimes	☐ Often	☐ Always
6. My child eats the same food with the rest of the family	☐ Never	☐ Rarely	☐ Sometimes	☐ Often	☐ Always
7. My child eats at almost the same time every day	☐ Never	☐ Rarely	☐ Sometimes	☐ Often	☐ Always
8. My child eats the same amount of meals every day	☐ Never	☐ Rarely	☐ Sometimes	☐ Often	☐ Always
9. My child eats autonomously (without an adult’s help)	☐ Never	☐ Rarely	☐ Sometimes	☐ Often	☐ Always
10. My child sits at the table during the meal	☐ Never	☐ Rarely	☐ Sometimes	☐ Often	☐ Always
11. My child watches TV while eating	☐ Never	☐ Rarely	☐ Sometimes	☐ Often	☐ Always
12. My child brings toys (books, tablets, etc.) at the table during the meal	☐ Never	☐ Rarely	☐ Sometimes	☐ Often	☐ Always
13. My child decides how much he/she is going to eat at every meal	☐ Never	☐ Rarely	☐ Sometimes	☐ Often	☐ Always
14. My child decides when he/she is going to eat every meal	☐ Never	☐ Rarely	☐ Sometimes	☐ Often	☐ Always
15. My child decides what he/she is going to eat from the food I have served	☐ Never	☐ Rarely	☐ Sometimes	☐ Often	☐ Always
16. My child informs me or conveys to me that he/she is hungry	☐ Never	☐ Rarely	☐ Sometimes	☐ Often	☐ Always
17. My child informs me or conveys it when he/she is full	☐ Never	☐ Rarely	☐ Sometimes	☐ Often	☐ Always
18. I decide when it is time for my child to eat food	☐ Never	☐ Rarely	☐ Sometimes	☐ Often	☐ Always
19. I decide when it is time for my child to eat a snack	☐ Never	☐ Rarely	☐ Sometimes	☐ Often	☐ Always
20. I decide which food my child is going to eat	☐ Never	☐ Rarely	☐ Sometimes	☐ Often	☐ Always
21. I decide which snacks my child is going to eat	☐ Never	☐ Rarely	☐ Sometimes	☐ Often	☐ Always
22. We eat at the table	☐ Never	☐ Rarely	☐ Sometimes	☐ Often	☐ Always
23. We eat at least one meal a day all of us together (or almost all of us)	☐ Never	☐ Rarely	☐ Sometimes	☐ Often	☐ Always
24. We eat at almost the same time every day	☐ Never	☐ Rarely	☐ Sometimes	☐ Often	☐ Always
