# Supplementary material for: Mealtime Environment and Control of Food Intake in Healthy Children and in Children with Gastrointestinal Diseases
Source: Children (Basel). 2021 Jan 23;8(2):77. doi: 10.3390/children8020077 (PMC7912501; doi:10.3390/children8020077)
Supplement: Supplementary file 1 [file children-08-00077-s001.pdf]

Table S0. Frequency distribution of the answers to the scale questions in the two groups.

		Initialcoding (5-point)						Condensedcoding (2-point)[α]		
		Never	Rarely	Sometimes	Often	Veryoften	*p*	Relatively	Relatively	*p*
Q1	Control	0.5%	3.8%	8.4%	19.1%	68.2%	0.076	**12.7%**	**87.3%**	**0.023**
	Case	0.7%	7.8%	11.3%	22.7%	57.4%		**19.9%**	**80.1%**	
Q2	Control	1.1%	9.9%	15.1%	25.8%	48.0%	0.803	26.2%	73.8%	0.473
	Case	2.1%	11.3%	15.6%	27.0%	44.0%		29.1%	70.9%	
Q3	Control	0.8%	5.3%	8.9%	30.0%	55.0%	0.477	15.0%	85.0%	0.298
	Case	2.1%	7.1%	9.2%	31.2%	50.4%		18.4%	81.6%	
Q4	Control	**4.1%**	**9.8%**	**33.3%**	**38.6%**	**14.2%**	**0.030**	47.1%	52.9%	0.052
	Case	**7.1%**	**17.0%**	**31.9%**	**29.8%**	**14.2%**		56.0%	44.0%	
Q5	Control	**0.4%**	**2.9%**	**13.7%**	**26.0%**	**56.9%**	**0.025**	17.0%	83.0%	0.069
	Case	**1.4%**	**7.1%**	**14.9%**	**29.8%**	**46.8%**		23.4%	76.6%	
Q6	Control	**0.6%**	**3.0%**	**5.1%**	**16.0%**	**75.2%**	**<0.001**	**8.8%**	**91.2%**	**<0.001**
	Case	**1.4%**	**6.4%**	**10.6%**	**24.8%**	**56.7%**		**18.4%**	**81.6%**	
Q7	Control	0.3%	0.6%	3.8%	22.6%	72.7%	0.776	4.7%	95.3%	0.621
	Case	0.7%	1.4%	3.5%	22.7%	71.6%		5.7%	94.3%	
Q8	Control	**0.5%**	**1.9%**	**11.4%**	**35.5%**	**50.7%**	**0.041**	13.9%	86.1%	0.567
	Case	**2.8%**	**0.7%**	**8.5%**	**39.0%**	**48.9%**		12.1%	87.9%	
Q9	Control	**0.3%**	**3.4%**	**10.8%**	**21.6%**	**63.9%**	**<0.001**	**14.5%**	**85.5%**	**<0.001**
	Case	**4.3%**	**13.5%**	**20.6%**	**22.0%**	**39.7%**		**38.3%**	**61.7%**	
Q10	Control	**0.4%**	**1.9%**	**6.6%**	**21.2%**	**69.9%**	**<0.001**	**8.9%**	**91.1%**	**<0.001**
	Case	**2.8%**	**6.4%**	**12.8%**	**17.7%**	**60.3%**		**22.0%**	**78.0%**	
Q11	Control	**13.1%**	**21.1%**	**34.9%**	**22.9%**	**8.0%**	**0.031**	**69.1%**	**30.9%**	**0.010**
	Case	**9.2%**	**18.4%**	**30.5%**	**26.2%**	**15.6%**		**58.2%**	**41.8%**	
Q12	Control	**39.9%**	**28.7%**	**17.2%**	**10.5%**	**3.7%**	**0.007**	**85.8%**	**14.2%**	**0.001**
	Case	36.9%	22.0%	15.6%	16.3%	9.2%		74.5%	25.5%	
Q13	Control	2.5%	10.7%	22.5%	30.1%	34.2%	0.075	35.7%	64.3%	0.668
	Case	6.4%	10.6%	20.6%	23.4%	39.0%		37.6%	62.4%	
Q14	Control	16.4%	32.8%	28.5%	16.6%	5.7%	0.378	77.6%	22.4%	0.521
	Case	13.5%	32.6%	29.1%	14.9%	9.9%		75.2%	24.8%	
Q15	Control	8.6%	21.2%	24.8%	26.4%	18.9%	0.798	54.6%	45.4%	0.760
	Case	11.3%	19.9%	24.8%	23.4%	20.6%		56.0%	44.0%	
Q16	Control	**1.8%**	**3.3%**	**8.8%**	**22.5%**	**63.7%**	**0.026**	**13.9%**	**86.1%**	**0.002**
	Case	**2.8%**	**5.7%**	**15.6%**	**24.1%**	**51.8%**		24.1%	75.9%	
Q17	Control	0.4%	0.8%	1.5%	16.1%	81.2%	0.398	2.7%	97.3%	0.710
	Case	0%	0%	2.1%	21.3%	76.6%		2.1%	97.9%	
Q18	Control	1.8%	4.7%	17.3%	36.7%	39.4%	0.203	23.9%	76.1%	0.121
	Case	2.9%	9.3%	17.9%	32.9%	37.1%		30.0%	70.0%	
Q19	Control	3.7%	8.7%	24.4%	33.8%	29.4%	0.610	36.8%	63.2%	0.491
	Case	2.2%	12.3%	25.4%	31.9%	28.3%		39.9%	60.1%	
Q20	Control	1.8%	4.1%	9.3%	32.4%	52.4%	0.181	15.2%	84.8%	0.384
	Case	2.9%	1.4%	13.8%	27.5%	54.3%		18.1%	81.9%	
Q21	Control	1.4%	4.7%	18.8%	35.5%	39.7%	0.186	24.9%	75.1%	0.270
	Case	2.9%	5.7%	20.7%	25.7%	45.0%		29.3%	70.7%	
Q22	Control	**12.3%**	**31.2%**	**33.7%**	**17.4%**	**5.5%**	**0.001**	77.1%	22.9%	0.712
	Case	**16.4%**	**34.3%**	**25.0%**	**10.7%**	**13.6%**		75.7%	24.3%	
Q23	Control	6.6%	8.7%	18.2%	30.1%	36.4%	0.409	33.5%	66.5%	0.098
	Case	5.7%	7.1%	13.6%	29.3%	44.3%		26.4%	73.6%	
Q24	Control	0.8%	6.9%	17.6%	34.0%	40.7%	0.250	25.3%	74.7%	0.521
	Case	2.9%	7.9%	17.1%	35.0%	37.1%		27.9%	72.1%	

[α]Note: Condensed coding was applied as follows: Relatively rare: "Never"/"Rarely"/"Sometimes"& Relatively often: "Often" / "Very often or always". p-value for chi-square test. *p*-values below 0.05 are shown in bold.Conclusions (in-text, Results section) are based on condensed coding.

Table S1. The parental practices in association with the child's sex, birth order and the existence of sib-lings. Group: healthy children.

		Child'ssex				OnlyChild				First born			
		Male	Female	Chi square	*p*	No	Yes	Chi square	*p*	No	Yes	Chi square	*p*
		N = 393	*N* = 394			*N* = 604	*N* = 183			*N* = 376	*N* = 411		
Q1	Relativelyrare	14.2%	11.2%	1.685	0.194	11.8%	15.8%	2.120	0.145	10.4%	14.8%	3.536	0.060
	Relativelyoften	85.8%	88.8%			88.2%	84.2%			89.6%	85.2%		
Q2	Relativelyrare	27.5%	24.9%	0.692	0.405	26.2%	26.2%	0,001	0.985	24.7%	27.5%	0.774	0.379
	Relativelyoften	72.5%	75.1%			73.8%	73.8%			75.3%	72.5%		
Q3	Relativelyrare	15.3%	14.7%	0.046	0.830	14.7%	15.8%	0.136	0.712	14.4%	15.6%	0.226	0.635
	Relativelyoften	84.7%	85.3%			85.3%	84.2%			85.6%	84.4%		
Q4	Relativelyrare	**52.2%**	**42.1%**	**7.945**	**0.005**	48.0%	44.3%	0.793	0.373	46.0%	48.2%	0.369	0.543
	Relativelyoften	**47.8%**	**57.9%**			52.0%	55.7%			54.0%	51.8%		
Q5	Relativelyrare	18.8%	15.2%	1.806	0.179	**15.4%**	**22.4%**	**4.881**	**0.027**	14.6%	19.2%	2.933	0.087
	Relativelyoften	81.2%	84.8%			**84.6%**	**77.6%**			85.4%	80.8%		
Q6	Relativelyrare	8.9%	8.6%	0.019	0.891	**7.6%**	**12.6%**	**4.306**	**0.038**	7.7%	9.7%	1.001	0.317
	Relativelyoften	91.1%	91.4%			**92.4%**	**87.4%**			92.3%	90.3%		
Q7	Relativelyrare	4.1%	5.3%	0.696	0.404	4.5%	5.5%	0.310	0.578	3.7%	5.6%	1.537	0.215
	Relativelyoften	95.9%	94.7%			95.5%	94.5%			96.3%	94.4%		
Q8	Relativelyrare	**11.2%**	**16.5%**	**4.635**	**0.031**	13.1%	16.4%	1.293	0.256	11.4%	16.1%	3.516	0.061
	Relativelyoften	**88.8%**	**83.5%**			86.9%	83.6%			88.6%	83.9%		
Q9	Relativelyrare	**17.8%**	**11.2%**	**7.012**	**0.008**	**11.9%**	**23.0%**	**13.795**	**<0.001**	**10.9%**	**17.8%**	**7.454**	**0.006**
	Relativelyoften	**82.2%**	**88.8%**			**88.1%**	**77.0%**			**89.1%**	**82.2%**		
Q10	Relativelyrare	**10.9%**	**6.9%**	**4.059**	**0.044**	**7,0%**	**15.3%**	**12.075**	**0.001**	**5.6%**	**11.9%**	**9.731**	**0.002**
	Relativelyoften	**80.1%**	**93.1%**			**93,0%**	**84.7%**			**94.4%**	**88.1%**		
Q11	Relativelyrare	66.2%	72.1%	3.235	0.072	70.0%	66.1%	1.008	0.315	**73.7%**	**65.0%**	**6.974**	**0.008**
	Relativelyoften	33.8%	27.9%			30.0%	33.9%			**26.3%**	**35.0%**		
Q12	Relativelyrare	85.0%	86.5%	0.393	0.531	86.3%	84.2%	0.510	0.475	**89.9%**	**82.0%**	**10.036**	**0.002**
	Relativelyoften	15.0%	13.5%			13.7%	15.8%			**10.1%**	**18.0%**		
Q13	Relativelyrare	37.4%	34.0%	0.988	0.320	35.6%	36.1%	0.013	0.908	36.4%	35.0%	0.168	0.682
	Relativelyoften	62.6%	66.0%			64.4%	63.9%			63.6%	65.0%		
Q14	Relativelyrare	80.2%	75.1%	2.862	0.091	77.3%	78.7%	0.152	0.697	79.0%	76.4%	0.759	0.384
	Relativelyoften	19.8%	24.9%			22.7%	21.3%			21.0%	23.6%		
Q15	Relativelyrare	55.7%	53.6%	0.374	0.541	54.0%	56.8%	0.463	0.496	55.9%	53.5%	0.428	0.513

	Relativelyoften	44.3%	46.4%			46.0%	43.2%			44.1%	46.5%		
Q16	Relativelyrare	14.0%	13.7%	0.014	0.906	**11.3%**	**22.4%**	**14.624**	**<0.001**	**10.6%**	**16.8%**	**6.224**	**0.013**
	Relativelyoften	86.0%	86.3%			**88.7%**	**77.6%**			**89.4%**	**83.2%**		
Q17	Relativelyrare	**3.8%**	**1.5%**	**3.986**	**0.046**	2.6%	2.7%	0.004	0.951	2.7%	2.7%	0.001	0.988
	Relativelyoften	**96.2%**	**98.5%**			97.4%	97.3%			97.3%	97.3%		
Q18	Relativelyrare	22.2%	25.5%	1.187	0.276	23.1%	26.5%	0.922	0.337	21.5%	26.0%	2.122	0.145
	Relativelyoften	77.8%	74.5%			76.9%	73.5%			78.5%	74.0%		
Q19	Relativelyrare	36.5%	37.1%	0.031	0.861	36.3%	38.3%	0.242	0.623	35.9%	37.6%	0.239	0.625
	Relativelyoften	63.5%	62.9%			63.7%	61.7%			64.1%	62.4%		
Q20	Relativelyrare	15.3%	15.1%	0.007	0.933	15.6%	13.8%	0.351	0.549	16.0%	14.5%	0.359	0.549
	Relativelyoften	84.7%	84.9%			84.4%	86.2%			84.0%	85.5%		
Q21	Relativelyrare	24.0%	25.8%	0.334	0.563	25.5%	22.7%	0.621	0.431	26.6%	23.3%	1.148	0.284
	Relativelyoften	76.0%	74.2%			74.5%	77.3%			73.4%	76.7%		
Q22	Relativelyrare	75.2%	79.1%	1.680	0.195	78.4%	72.8%	2.521	0.112	79.3%	75.2%	1.837	0.175
	Relativelyoften	24.8%	20.9%			21.6%	27.2%			20.7%	24.8%		
Q23	Relativelyrare	**29.6%**	**37.5%**	**5.499**	**0.019**	32.7%	36.5%	0.899	0.343	33.0%	34.1%	0.104	0.747
	Relativelyoften	**70.4%**	**62.5%**			67.3%	63.5%			67.0%	65.9%		
Q24	Relativelyrare	23.0%	27.6%	2.252	0.133	**27.1%**	**19.3%**	**4.412**	**0.036**	27.7%	23.0%	2.279	0.131
	Relativelyoften	77.0%	72.4%			**72.9%**	**80.7%**			72.3%	77.0%		

Table S2. The parental practices in association with the child’s age group, parent’s sex, and child-parent sex concurrence. Group: healthy children.

		**Child'sagegroup**				**Parent'ssex**				**Sexconcordance**			
		**≤5 years**	**>5 years**	**Chi square**	*p*	**Female**	**Male**	**Chi square**	*p*	**No**	**Yes**	**Chi square**	*p*
		***N* = 367**	***N* = 420**			***N* = 730**	***N* = 57**			***N* = 399**	***N* = 388**		
Q1	Relativelyrare	12.5%	12.9%	0.018	0.892	13.2%	7.0%	1.793	0.181	14.9%	10.5%	3.468	0.063
	Relativelyoften	87.5%	87.1%			86.8%	93.0%			85.1%	89.5%		
Q2	Relativelyrare	27.0%	25.5%	0.228	0.633	26.6%	21.1%	0.835	0.361	28.4%	24.1%	1.874	0.171
	Relativelyoften	73.0%	74.5%			73.4%	78.9%			71.6%	75.9%		
Q3	Relativelyrare	13.9%	16.0%	0.650	0.420	14.9%	15.8%	0.031	0.861	16.2%	13.8%	0.928	0.335
	Relativelyoften	86.1%	84.0%			85.1%	84.2%			83.8%	86.2%		
Q4	Relativelyrare	48.0%	46.4%	0.183	0.668	46.3%	57.9%	2.852	0.091	**42.9%**	**51.5%**	**5.960**	**0.015**
	Relativelyoften	52.0%	53.6%			53.7%	42.1%			**57.1%**	**48.5%**		
Q5	Relativelyrare	16.3%	17.6%	0.224	0.636	17.0%	17.5%	0.012	0.914	18.0%	16.0%	0.558	0.455
	Relativelyoften	83.7%	82.4%			83.0%	82.5%			82.0%	84.0%		
Q6	Relativelyrare	8.7%	8.8%	0.002	0.964	8.9%	7.0%	0.235	0.628	9.0%	8.5%	0.061	0.804
	Relativelyoften	91.3%	91.2%			91.1%	93.0%			91.0%	91.5%		
Q7	Relativelyrare	5.4%	4.0%	0.859	0.354	4.5%	7.0%	0.736	0.391	3.6%	5.8%	2.041	0.153
	Relativelyoften	94.6%	96.0%			95.5%	93.0%			96.4%	94.2%		
Q8	Relativelyrare	13.6%	14.0%	0.029	0.864	14.2%	8.8%	1.328	0.249	11.6%	16.0%	3.253	0.071
	Relativelyoften	86.4%	86.0%			85.8%	91.2%			88.4%	84.0%		
Q9	Relativelyrare	**21.0%**	**8.8%**	**23.423**	**<0.001**	14.4%	15.8%	0.084	0.771	16.2%	12.8%	1.896	0.169
	Relativelyoften	**79.0%**	**91.2%**			85.6%	84.2%			83.8%	87.2%		
Q10	Relativelyrare	10.1%	7.9%	1.196	0.274	8.8%	10.5%	0.202	0.653	10.6%	7.3%	2.642	0.104
	Relativelyoften	89.9%	92.1%			91.2%	89.5%			89.4%	92.7%		
Q11	Relativelyrare	71.4%	67.1%	1.655	0.198	69.2%	68.4%	0.014	0.905	**65.2%**	**72.9%**	**5.502**	**0.019**
	Relativelyoften	28.6%	32.9%			30.8%	31.6%			**34.8%**	**27.1%**		
Q12	Relativelyrare	84.7%	86.7%	0.595	0.441	85.8%	86.0%	0.002	0.965	85.3%	86.2%	0.132	0.716
	Relativelyoften	15.3%	13.3%			14.2%	14.0%			14.7%	13.8%		
Q13	Relativelyrare	**31.6%**	**39.3%**	**5.030**	**0.025**	36.0%	31.6%	0.456	0.500	36.9%	34.6%	0.441	0.507
	Relativelyoften	**68.4%**	**60.7%**			64.0%	68.4%			63.1%	65.4%		
Q14	Relativelyrare	78.5%	76.9%	0.278	0.598	77.5%	78.9%	0.061	0.805	78.9%	76.4%	0.666	0.414
	Relativelyoften	21.5%	23.1%			22.5%	21.1%			21.1%	23.6%		
Q15	Relativelyrare	52.3%	56.7%	1.496	0.221	54.8%	52.6%	0.100	0.752	55.4%	53.9%	0.185	0.667

	Relativelyoften	47.7%	43.3%			45.2%	47.4%			44.6%	46.1%		
Q16	Relativelyrare	14.4%	13.3%	0.202	0.653	**13.2%**	**22.8%**	**4.132**	**0.042**	13.9%	13.8%	0.003	0.957
	Relativelyoften	85.6%	86.7%			**86.8%**	**77.2%**			86.1%	86.2%		
Q17	Relativelyrare	2.5%	2.9%	0.124	0.725	2.5%	5.3%	1.593	0.207	**4.1%**	**1.3%**	**6.241**	**0.012**
	Relativelyoften	97.5%	97.1%			97.5%	94.7%			**95.9%**	**98.7%**		
Q18	Relativelyrare	24.7%	23.2%	0.244	0.621	**22.8%**	**36.2%**	**5.711**	**0.017**	22.2%	25.4%	1.118	0.290
	Relativelyoften	75.3%	76.8%			**77.2%**	**63.2%**			77.8%	74.6%		
Q19	Relativelyrare	34.9%	38.4%	1.047	0.306	37.1%	33.3%	0.314	0.575	35.7%	37.9%	0.415	0.520
	Relativelyoften	65.1%	61.6%			62.9%	66.7%			64.3%	62.1%		
Q20	Relativelyrare	14.3%	16.0%	0.439	0.508	14.7%	21.1%	1.635	0.201	15.0%	15.4%	0.026	0.871
	Relativelyoften	85.7%	84.0%			85.3%	78.9%			85.0%	84.6%		
Q21	Relativelyrare	**21.4%**	**27.9%**	**4.484**	**0.034**	24.2%	33.3%	2.355	0.125	24.5%	25.2%	0.043	0.836
	Relativelyoften	**78.6%**	**72.1%**			75.8%	66.7%			75.5%	74.8%		
Q22	Relativelyrare	74.7%	79.2%	2.248	0.134	77.0%	78.9%	0.114	0.736	76.9%	77.3%	0.017	0.897
	Relativelyoften	25.3%	20.8%			23.0%	21.1%			23.1%	22.7%		
Q23	Relativelyrare	34.8%	32.5%	0.478	0.490	32.9%	42.1%	2.020	0.155	**29.5%**	**37.5%**	**5.731**	**0.017**
	Relativelyoften	65.2%	67.5%			67.1%	57.9%			**70.5%**	**62.5%**		
Q24	Relativelyrare	24.5%	26.0%	0.252	0.616	24.5%	35.1%	3.125	0.077	26.4%	24.2%	0.521	0.470
	Relativelyoften	75.5%	74.0%			75.5%	64.9%			73.6%	75.8%		

Table S3. The parental practices in association with the parent's age, education and employment status. Group: healthy children.

		Parent'sagegroup				Parent'seducation				WorkingParent			
		<40 years	≥40 years	Chi square	*p*	≤12 years	>12 years	Chi square	*p*	No	Yes	Chi square	*p*
		***N* = 581**	***N* = 206**			***N* = 362**	***N* = 425**			***N* = 235**	***N* = 552**		
Q1	Relativelyrare	12.7%	12.6%	0.002	0.966	**9.8%**	**15.1%**	**4.609**	**0.032**	11.9%	13.0%	0.189	0.664
	Relativelyoften	87.3%	87.4%			**90.1%**	**84.9%**			88.1%	87.0%		
Q2	Relativelyrare	25.8%	27.2%	0.147	0.701	**21.3%**	**30.4%**	**8.345**	**0.004**	23.0%	27.5%	1.772	0.183
	Relativelyoften	74.2%	72.8%			**78.7%**	**69.6%**			77.0%	72.5%		
Q3	Relativelyrare	15.5%	84.5%	0.430	0.512	15.2%	14.8%	0.021	0.885	11.5%	16.5%	3.228	0.072
	Relativelyoften	13.6%	86.4%			84.8%	85.2%			88.5%	83.5%		
Q4	Relativelyrare	47.8%	45.1%	0.446	0.504	44.2%	49.6%	2.329	0.127	**54.9%**	**43.8%**	**8.081**	**0.004**
	Relativelyoften	52.2%	54.9%			55.8%	50.4%			**45.1%**	**56.2%**		
Q5	Relativelyrare	15.5%	21.4%	3.708	0.054	**12.7%**	**20.7%**	**8.853**	**0.003**	13.2%	18.7%	3.488	0.062
	Relativelyoften	84.5%	78.6%			**87.3%**	**79.3%**			86.8%	81.3%		
Q6	Relativelyrare	8.6%	9.2%	0.072	0.788	9.7%	8.0%	0.680	0.409	9.4%	8.5%	0.148	0.701
	Relativelyoften	91.4%	90.8%			90.3%	92.0%			90.6%	91.5%		
Q7	Relativelyrare	4.5%	5.3%	0.254	0.614	**6.6%**	**3.1%**	**5.564**	**0.027**	5.1%	4.5%	0.123	0.726
	Relativelyoften	95.5%	94.7%			**93.4%**	**96.9%**			94.9%	95.5%		
Q8	Relativelyrare	15.0%	10.7%	2.351	0.125	**18.8%**	**9.6%**	**13.769**	**<0.001**	17.4%	12.3%	3.633	0.057
	Relativelyoften	85.0%	89.3%			**81.2%**	**90.4%**			82.6%	87.7%		
Q9	Relativelyrare	14.6%	14.1%	0.037	0.847	13.3%	15.5%	0.813	0.367	13.9%	15.7%	0.429	0.513
	Relativelyoften	85.4%	85.9%			86.7%	84.5%			86.1%	84.3%		
Q10	Relativelyrare	8.6%	9.7%	0.228	0.633	7.5%	10.1%	1.706	.192	10.2%	8.3%	0.718	0.397
	Relativelyoften	91.4%	90.3%			92.5%	89.5%			89.8%	91.7%		
Q11	Relativelyrare	70.4%	65.5%	1.684	0.194	66.9%	71.1%	1.622	.203	69.9%	67.2%	0.560	0.454
	Relativelyoften	29.6%	34.5%			33.1%	28.9%			30.1%	32.8%		
Q12	Relativelyrare	85.0%	87.9%	1.004	0.316	83.4%	87.8%	3.016	0.082	82.6%	87.1%	2.838	0.092
	Relativelyoften	15.0%	12.1%			16.2%	12.2%			17.4%	12.9%		
Q13	Relativelyrare	35.8%	35.4%	0.009	0.925	38.4%	33.4%	2.117	0.146	38.7%	34.4%	1.330	0.249
	Relativelyoften	64.2%	64.6%			61.6%	66.6%			61.3%	65.6%		
Q14	Relativelyrare	78.0%	76.7%	0.141	0.707	77.1%	78.1%	0.123	0.726	78/.7%	77.2%	0.228	0.633
	Relativelyoften	22.0%	23.3%			22.9%	21.9%			21.3%	22.8%		
Q15	Relativelyrare	55.4%	52.4%	0.550	0.458	55.5%	53.9%	0.213	0.645	54.0%	54.9%	0.048	0.827

	Relativelyoften	44.6%	47.6%			44.5%	46.1%			46.0%	45.1%		
Q16	Relativelyrare	14.3%	12.6%	0.353	0.552	14.1%	13.6%	0.032	0.858	12.8%	14.3%	0.330	0.566
	Relativelyoften	85.7%	87.4%			85.9%	86.4%			87.2%	85.7%		
Q17	Relativelyrare	3.1%	1.5%	1.578	0.209	3.0%	2.4%	0.354	0.552	**0.9%**	**3.4%**	**4.260**	**0.039**
	Relativelyoften	96.9%	98.5%			97.0%	97.6%			**99.1%**	**96.6%**		
Q18	Relativelyrare	24.2%	22.9%	0.131	0.718	25.1%	22.7%	0.612	0.434	19.7%	25.6%	3.230	0.072
	Relativelyoften	75.8%	77.1%			74.9%	77.3%			80.3%	74.4%		
Q19	Relativelyrare	37.5%	34.6%	0.551	0.458	34.3%	38.9%	1.705	0.192	35.9%	37.2%	0.112	0.738
	Relativelyoften	62.5%	65.4%			65.7%	61.1%			64.1%	62.8%		
Q20	Relativelyrare	14.4%	17.6%	1.203	0.273	16.9%	13.7%	1.501	0.220	15.0%	15.3%	0.015	0.903
	Relativelyoften	85.6%	82.4%			83.1%	86.3%			85.0%	84.7%		
Q21	Relativelyrare	25.2%	23.9%	0.140	0.709	26.0%	23.9%	0.431	0.511	22.6%	25.8%	0.882	0.348
	Relativelyoften	74.8%	76.1%			74.0%	76.1%			77.4%	74.2%		
Q22	Relativelyrare	77.0%	77.6%	0.028	0.867	79.2%	75.4%	1.651	0.199	80.3%	75.8%	1.941	0.164
	Relativelyoften	23.0%	22.4%			20.8%	24.6%			19.7%	24.2%		
Q23	Relativelyrare	33.0%	35.1%	0.309	0.578	32.6%	34.4%	0.272	0.602	28.6%	35.6%	3.612	0.057
	Relativelyoften	67.0%	64.9%			67.4%	65.6%			71.4%	64.4%		
Q24	Relativelyrare	25.6%	24.4%	0.118	0.731	**21.8%**	**28.3%**	**4.276**	**0.039**	26.9%	24.6%	0.473	0.492
	Relativelyoften	74.4%	75.6%			**78.2%**	**71.7%**			73.1%	75.4%		

Table S4. The parental practices in association with the child's sex, birth order and the existence of sib-lings. Group: Children with gastrointestinal diseases.

		Child'ssex				OnlyChild				First born			
		Male	Female	Chi square	*p*	No	Yes	Chi square	*p*	No	Yes	Chi square	*p*
		N **= 77**	***N*** **= 64**			***N*** **= 95**	***N*** **= 46**			***N*** **= 50**	***N*** **= 91**		
Q1	Relativelyrare	14.3%	26.6%	3.310	0.069	18.9%	21.7%	0.152	0.697	18.0%	20.9%	0.168	0.682
	Relativelyoften	85.7%	73.4%			81.1%	78.3%			82.0%	79.1%		
Q2	Relativelyrare	34.4%	24.7%	1.594	0.207	30.5%	26.1%	0.296	0.586	30.0%	28.6%	0.032	0.858
	Relativelyoften	65.6%	75.3%			69.5%	73.9%			70.0%	71.4%		
Q3	Relativelyrare	21.9%	15.6%	0.920	0.338	17.9%	19.6%	0.058	0.810	18.0%	18.7%	0.010	0.921
	Relativelyoften	78.1%	84.4%			82.1%	80.4%			82.0%	81.3%		
Q4	Relativelyrare	**64.9%**	**45.3%**	**5.462**	**0.019**	53.7%	60.9%	0.649	0.420	56.0%	56.0%	0.001	0.996
	Relativelyoften	**35.1%**	**54.7%**			46.3%	39.1%			44.0%	44.0%		
Q5	Relativelyrare	27.3%	18.8%	1.416	0.234	18.9%	32.6%	3.227	0.072	20.0%	25.3%	0.501	0.479
	Relativelyoften	72.7%	81.3%			81.1%	67.4%			80.0%	74.7%		
Q6	Relativelyrare	20.8%	15.6%	0.617	0.432	17.9%	19.6%	0.058	0.810	20.0%	17.6%	0.125	0.723
	Relativelyoften	79.2%	84.4%			82.1%	80.4%			80.0%	82.4%		
Q7	Relativelyrare	5.2%	6.3%	0.073	0.787	6.3%	4.3%	0.224	0.636	6.0%	5.5%	0.015	0.901
	Relativelyoften	94.8%	93.8%			93.7%	95.7%			94.0%	94.5%		
Q8	Relativelyrare	14.3%	9.4%	0.795	0.373	12.6%	10.9%	0.091	0.763	14.0%	11.0%	0.276	0.599
	Relativelyoften	85.7%	90.6%			87.4%	89.1%			86.0%	89.0%		
Q9	Relativelyrare	39.0%	37.5%	0.032	0.859	37.9%	39.1	0.020	0.887	46.0%	34.1%	1.945	0.163
	Relativelyoften	61.0%	62.5%			62.1%	60.9%			54.0%	65.9%		
Q10	Relativelyrare	24.7%	18.8%	0.715	0.398	17.9%	30.4%	2.841	0.092	22.0%	22.0%	0.001	0.998
	Relativelyoften	75.3%	81.3%			82.1%	69.6%			78.0%	78.0%		
Q11	Relativelyrare	57.1%	59.4%	0.072	0.789	56.8%	60.9%	0.207	0.649	60.0%	57.1%	0.108	0.742
	Relativelyoften	42.9%	40.6%			43.2%	39.1%			40.0%	42.9%		
Q12	Relativelyrare	**64.9%**	**85.9%**	**8.108**	**0.004**	78.9%	62.5%	3.073	0.080	80.0%	71.4%	1.247	0.264
	Relativelyoften	**35.1%**	**14.1%**			21.1%	34.8%			20.0%	28.6%		
Q13	Relativelyrare	41.6%	32.8%	1.140	0.286	**45.3%**	**21.7%**	**7.311**	**0.007**	46.0%	33.0%	2.336	0.126
	Relativelyoften	58.4%	67.2%			**54.7%**	**78.3%**			54.0%	67.0%		
Q14	Relativelyrare	68.8%	82.8%	3.661	0.056	76.8%	71.7%	0.432	0.511	78.0%	73.6%	0.331	0.565
	Relativelyoften	31.2%	17.2%			23.2%	28.3%			22.0%	26.4%		
Q15	Relativelyrare	54.5%	57.8%	0.151	0.697	57.9%	52.2%	0.412	0.521	64.0%	51.6%	1.998	0.157

	Relativelyoften	45.5%	42.2%			42.1%	47.8%			36.0%	48.4%		
Q16	Relativelyrare	23.4%	25.0%	0.050	0.822	24.2%	23.9%	0.001	0.969	24.0%	24.2%	0.001	0.981
	Relativelyoften	76.6%	75.0%			75.8%	76.1%			76.0%	75.8%		
Q17	Relativelyrare	1.3%	3.1%	0.560	0.454	3.2%	0.0%	1.484	0.223	2.0%	2.2%	0.006	0.938
	Relativelyoften	98.7%	96.9%			96.8%	100.0%			98.0%	97.8%		
Q18	Relativelyrare	32.5%	27.0%	0.496	0.481	32.6%	24.4%	0.975	0.324	28.0%	31.1%	0.148	0.700
	Relativelyoften	67.5%	73.0%			67.4%	75.6%			72.0%	68.9%		
Q19	Relativelyrare	39.5%	40.3%	0.010	0.919	38.7%	42.2%	0.156	0.693	40.8%	39.3%	0.029	0.864
	Relativelyoften	60.5%	59.7%			61.3%	57.8%			59.2%	60.7%		
Q20	Relativelyrare	21.1%	14.5%	0.983	0.321	15.1%	24.4%	1.803	0.179	18.4%	18.0%	0.003	0.955
	Relativelyoften	78.9%	85.5%			84.9%	75.6%			81.6%	82.0%		
Q21	Relativelyrare	31.6%	26.6%	0.422	0.516	27.7%	32.6%	0.365	0.546	32.7%	27.5%	0.413	0.521
	Relativelyoften	68.4%	73.4%			72.3%	67.4%			67.3%	72.5%		
Q22	Relativelyrare	73.7%	78.1%	0.373	0.542	**81.9%**	**63.0%**	**5.982**	**0.014**	77.6%	74.7%	0.138	0.710
	Relativelyoften	26.3%	21.9%			**18.1%**	**37.0%**			22.4%	25.3%		
Q23	Relativelyrare	27.6%	25.0%	0.124	0.725	24.5%	30.4%	0.566	0.452	24.5%	27.5%	0.146	0.703
	Relativelyoften	72.4%	75.0%			75.5%	69.6%			75.5%	72.5%		
Q24	Relativelyrare	26.3%	29.7%	0.197	0.658	31.9%	19.6%	2.344	0.126	36.7%	23.1%	2.956	0.086
	Relativelyoften	73.7%	70.3%			68.1%	80.4%			63.3%	76.9%		

Table S5. The parental practices in association with the child’s age group, parent’s sex, and child-parent sex concurrence. Group: Children with gastrointestinal diseases.

		Child'sagegroup				Parent'ssex				Sexconcordance			
		≤5 years	>5 years	Chi square	*p*	Female	Male	Chi square	*p*	No	Yes	Chi square	*p*
		N **=99**	***N*** **= 42**			***N*** **= 136**	***N*** **= 5**			***N*** **= 80**	***N*** **= 61**		
Q1	Relativelyrare	21.2%	16.7%	0.383	0.536	19.9%	20.0%	0.001	0.994	15,0%	26.2%	2.742	0.098
	Relativelyoften	78.8%	83.3%			80.1%	80.0%			85,0%	73.8%		
Q2	Relativelyrare	33.3%	19.0%	2.918	0.088	29.4%	20.0%	0.207	0.649	25.0%	34.4%	1.491	0.222
	Relativelyoften	66.7%	81.0%			70.6%	80.0%			75.0%	65.6%		
Q3	Relativelyrare	20.2%	14.3%	0.686	0.407	18.4%	20.0%	0.008	0.927	16.3%	21.3%	0.590	0.443
	Relativelyoften	79.8%	85.7%			81.6%	80.0%			83.8%	78.7%		
Q4	Relativelyrare	59.6%	47.6%	1.717	0.190	55.1%	80.0%	1.209	0.272	**67.5%**	**41,0%**	**9.878**	**0.002**
	Relativelyoften	40.4%	52.4%			44.9%	20.0%			**32.5%**	**59,0%**		
Q5	Relativelyrare	**29.3%**	**9.5%**	**6.429**	**0.011**	23.5%	20.0%	0.034	0.855	27.5%	18.0%	1.730	0.188
	Relativelyoften	**70.7%**	**90.5%**			76.5%	80.0%			72.5%	82.0%		
Q6	Relativelyrare	**24.2%**	**4.8%**	**7.441**	**0.006**	18.4%	20.0%	0.008	0.927	21.3%	14.8%	0.971	0.324
	Relativelyoften	**75.8%**	**95.2%**			81.6%	80.0%			78.8%	85.2%		
Q7	Relativelyrare	5.1%	7.1%	0.241	0.623	5.9%	0.0%	0.312	0.577	5.0%	6.6%	0.157	0.692
	Relativelyoften	94.9%	92.9%			94.1%	100.0%			95.0%	93.4%		
Q8	Relativelyrare	12.1%	11.9%	0.001	0.971	11.8%	20.0%	0.308	0.579	15.0%	8.2%	1.511	0.219
	Relativelyoften	87.9%	88.1%			88.2%	80.0%			85.0%	91.8%		
Q9	Relativelyrare	**49.5%**	**11.9%**	**17.634**	**<0.001**	38.2%	40.0%	0.006	0.936	40.0%	36.1%	0.227	0.634
	Relativelyoften	**50.5%**	**88.1%**			61.8%	60.0%			60.0%	63.9%		
Q10	Relativelyrare	28.3%	7.1%	7.683	**0.006**	21.3%	40.0%	0.981	0.322	23.8%	19.7%	0.336	0.562

	Relativelyoften	71.7%	92.9%			78.7%	60.0%			76.3%	80.3%		
Q11	Relativelyrare	59.6%	54.8%	0.283	0.595	57.4%	80.0%	1.016	0.313	57.5%	59.0%	0.033	0.856
	Relativelyoften	40.4%	45.2%			42.6%	20.0%			42.5%	41.0%		
Q12	Relativelyrare	71.7%	81.0%	1.323	0.250	74.3%	80.0%	0.083	0.773	**65,0%**	**86.9%**	**8.719**	**0.003**
	Relativelyoften	28.3%	19.0%			25.7%	20.0%			**35,0%**	**13.1%**		
Q13	Relativelyrare	34.3%	45.2%	1.492	0.222	36.8%	60.0%	1.110	0.292	41.3%	32.8%	1.057	0.304
	Relativelyoften	65.7%	54.8%			63.2%	40.0%			58.8%	67.2%		
Q14	Relativelyrare	73.7%	78.6%	0.369	0.543	74.3%	100.0%	1.712	0.191	70.0%	82.0%	2.656	0.103
	Relativelyoften	26.3%	21.4%			25.7%	0.0%			30.0%	18.0%		
Q15	Relativelyrare	52.5%	64.3%	1.656	0.198	**54.4%**	**100.0%**	**4.068**	**0.044**	56.3%	55.7%	0.004	0.952
	Relativelyoften	47.5%	35.7%			**45.6%**	**0.0%**			43.8%	44.3%		
Q16	Relativelyrare	23.2%	26.2%	0.141	0.707	24.3%	20.0%	0.048	0.827	21.3%	27.9%	0.829	0.363
	Relativelyoften	76.8%	73.8%			75.7%	80.0%			78.8%	72.1%		
Q17	Relativelyrare	2.0%	2.4%	0.018	0.892	2.2%	0.0%	0.113	0.737	1.3%	3.5%	0.684	0.408
	Relativelyoften	98.0%	97.6%			97.8%	100.0%			98.8%	96.7%		
Q18	Relativelyrare	33.7%	21.4%	2.099	0.147	30.4%	20.0%	0.247	0.619	32.5%	26.7%	0.556	0.456
	Relativelyoften	66.3%	78.6%			69.6%	80.0%			67.5%	73.3%		
Q19	Relativelyrare	38.1%	43.9%	0.399	0.528	40.6%	20.0%	0.853	0.356	39.2%	40.7%	0.029	0.865
	Relativelyoften	61.9%	56.1%			59.4%	80.0%			60.8%	59.3%		
Q20	Relativelyrare	16.5%	22.0%	0.578	0.447	18.8%	0.0%	1.148	0.284	20.3%	15.3%	0.569	0.451
	Relativelyoften	83.5%	78.0%			81.2%	100.0%			79.7%	84.7%		
Q21	Relativelyrare	30.6%	26.2%	0.278	0.598	30.4%	0.0%	2.147	0.143	30.4%	27.9%	0.105	0.746

	Relativelyoften	69.4%	73.8%			69.6%	100.0%			69.6%	72.1%		
Q22	Relativelyrare	73.5%	81.0%	0.895	0.344	76.3%	60.0%	0.696	0.404	72.2%	80.3%	1.251	0.263
	Relativelyoften	26.5%	19.0%			23.7%	40.0%			27.8%	19l.7%		
Q23	Relativelyrare	26.5%	26.2%	0.002	0.967	27.4%	0.0%	1.863	0.172	26.6%	26.2%	0.002	0.963
	Relativelyoften	73.5%	73.8%			72.6%	100.0%			73.4%	73.8%		
Q24	Relativelyrare	27.6%	28.6%	0.015	0.902	27.4%	40.0%	0.380	0.537	25.3%	31.1%	0.583	0.445
	Relativelyoften	72.4%	71.4%			72.6%	60.0%			74.7%	68.9%		

Table S6. The parental practices in association with the parent's age, education and employment status. Group: Children with gastrointestinal diseases.

		Parent'sagegroup				Parent'seducation				WorkingParent			
		<40 years *N* = 109	≥40 years *N* = 32	Chi square	*p*	≤12 years *N* = 49	>12 years *N* = 92	Chi square	*p*	No ***N* = 84**	Yes ***N* = 57**	Chi square	*p*
Q1	Relativelyrare	22.0%	12.5%	1.408	**0.235**	16.3%	21.7%	0.589	0.443	14.0%	23.8%	2.039	0.153
	Relativelyoften	78.0%	87.5%			83.7%	78.3%			86.0%	76.2%		
Q2	Relativelyrare	32.1%	18.8%	2.141	0.143	22.4%	32.6%	1.600	0.206	**14,0%**	**39.3%**	**10.499**	**0.001**
	Relativelyoften	67.9%	81.3%			77.6%	67.4%			**86,0%**	**60.7%**		
Q3	Relativelyrare	21.1%	9.4%	2.262	0.133	20.4%	17.4%	0.193	0.660	15.8%	20.2%	0.447	0.504
	Relativelyoften	78.9%	90.6%			79.6%	82.6%			84.2%	79.8%		
Q4	Relativelyrare	59.6%	43.8%	2.533	0.111	55.1%	56.5%	0.026	0.872	59.6%	53.6%	0.509	0.476
	Relativelyoften	40.4%	56.3%			44.9%	43.5%			40.4%	46.4%		
Q5	Relativelyrare	26.6%	12.5%	2.746	0.098	24.5%	22.8%	0.049	0.824	19.3%	26.2%	0.900	0.343
	Relativelyoften	73.4%	87.5%			75.5%	77.2%			80.7%	73.8%		
Q6	Relativelyrare	19.3%	15.6%	0.218	0.641	24.5%	15.2%	1.828	0.176	19.3%	17.9%	0.047	0.829
	Relativelyoften	80.7%	84.4%			75.5%	84.8%			80.7%	82.1%		
Q7	Relativelyrare	6.4%	3.1%	0.502	0.478	6.1%	5.4%	0.028	0.867	6.0%	5.3%	0.030	0.862
	Relativelyoften	93.6%	96.9%			93.9%	94.6%			94.0%	94.7%		
Q8	Relativelyrare	13.8%	6.3%	1.316	0.251	16.3%	9.8%	1.291	0.256	12.3%	11.9%	0.005	0.946
	Relativelyoften	86.2%	93.8%			83.7%	90.2%			87.7%	88.1%		
Q9	Relativelyrare	42.2%	25,0%	3.098	0.078	36.7%	39.1%	0.078	0.781	45.6%	33.3%	2.167	0.141
	Relativelyoften	57.8%	75,0%			63.3%	60.9%			54.4%	66.7%		
Q10	Relativelyrare	23.9%	15.6%	0.976	0.323	22.4%	21.7%	0.009	0.923	26.3%	19.0%	1.046	0.306
	Relativelyoften	76.1%	84.4%			77.6%	78.3%			73.7%	81.0%		
Q11	Relativelyrare	59.6%	53.1%	0.431	0.512	57.1%	58.7%	0.032	.859	57.9%	58.3%	0.003	0.959
	Relativelyoften	40.4%	46.9%			42.9%	41.3%			42.1%	41.7%		
Q12	Relativelyrare	70.6%	87.5%	3.697	0.054	77.6%	72.8%	0.375	.540	70.2%	77.4%	0.927	0.336
	Relativelyoften	29.4%	12.5%			22.4%	27.2%			29.8%	22.6%		
Q13	Relativelyrare	40.4%	28.1%	1.580	0.209	44.9%	33.7%	1.710	.191	43.9%	33.3%	1.604	0.205
	Relativelyoften	59.6%	71.9%			55.1%	66.3%			56.1%	66.7%		
Q14	Relativelyrare	71.6%	87.5%	3.368	0.066	73.5%	76.1%	0.117	.732	80.7%	71.4%	1.565	0.211
	Relativelyoften	28.4%	12.5%			26.5%	23.9%			19.3%	28.6%		
Q15	Relativelyrare	57.8%	50.0%	0.611	0.435	55.1%	56.5%	0.026	.872	63.2%	51.2%	1.974	0.160

	Relativelyoften	42.2%	50.0%			44.9%	43.5%			36.8%	48.8%		
Q16	Relativelyrare	22.0%	31.3%	1.152	0.283	22.4%	25.0%	0.114	.736	28.1%	21.4%	0.819	0.366
	Relativelyoften	78.0%	68.8%			77.6%	75.0%			71.9%	78.6%		
Q17	Relativelyrare	2.8%	0.0%	0.900	0.343	2.0%	2.2%	0.003	.958	1.8%	2.4%	0.064	0.800
	Relativelyoften	97.2%	100.0%			98.0%	97.8%			98.2%	97.6%		
Q18	Relativelyrare	33.3%	18.8%	2.500	0.114	30.6%	29.7%	0.013	.908	21.1%	36.1%	3.665	0.056
	Relativelyoften	66.7%	81.3%			69.4%	70.3%			78.9%	63.9%		
Q19	Relativelyrare	42.1%	32.3%	0.963	0.327	36.2%	41.8%	0.404	.525	35.7%	42.7%	0.674	0.412
	Relativelyoften	57.9%	67.7%			63.8%	58.2%			64.3%	57.3%		
Q20	Relativelyrare	15.9%	25.8%	1.594	0.207	20.8%	16.7%	0.366	.545	17.9%	18.3%	0.004	0.948
	Relativelyoften	84.1%	74.2%			79.2%	83.3%			82.1%	81.7%		
Q21	Relativelyrare	30.6%	25.0%	0.368	0.544	26.5%	30.8%	0.276	0.599	26.3%	31.3%	0.409	0.522
	Relativelyoften	69.4%	75.0%			73.5%	69.2%			73.7%	68.7%		
Q22	Relativelyrare	73.1%	84.4%	1.692	0.193	75.5%	75.8%	0.002	0.967	80.7%	72.3%	1.301	0.254
	Relativelyoften	26.9%	15.6%			24.5%	24.2%			19.3%	27.7%		
Q23	Relativelyrare	26.9%	25.0%	0.044	0.835	26.5%	26.4%	0.001	0.984	21.1%	30.1%	1.429	0.232
	Relativelyoften	73.1%	75.0%			73.5%	73.6%			78.9%	69.9%		
Q24	Relativelyrare	26.9%	31.3%	0.238	0.626	22.4%	30.8%	1.097	0.295	29.8%	26.5%	0.185	0.667
	Relativelyoften	73.1%	68.8%			77.6%	69.2%			70.2%	73.5%		

Table S7. Associations between items 1–24 and demographics as well with group. Two-layer chi-square.

Item	Child's sex	Only child	Firstborn	Child's age group (≤5 years vs > 5 years)	Parent's sex	Sex concordance	Parent's age group (<40 years vs ≥40 years)	Parent's education (≤12 years vs > 12 years)	Working parent
Q1	0.171 (.679)	2.680 (.102)	**4.224 (.040)**	0.269 (0.604)	1.834 (0.176)	1.076 (0.300)	0.380 (0.538)	**5.914 (0.015)**	0.768 (0.381)
Q2	0.080 (.777)	0.027 (.870)	0.774 (.379)	1.401 (0.237)	1.085 (0.298)	0.652(0.419)	0.052 (0.819)	**10.233 (0.001)**	**6.322 (0.012)**
Q3	0.029 (.866)	0.269 (.604)	0.319 (.572)	0.056 (0.812)	0.019 (0.890)	0.376 (0.540)	1.516 (0.218)	0.050 (0.823)	3.304 (0.069)
Q4	**12.667 (<.001)**	0.097 (.755)	0.540 (.463)	1.407 (0.236)	3.328 (0.068)	**12.638(<0.001)**	1.600 (0.206)	2.645 (0.104)	**9.166 (0.002)**
Q5	3.172 (.075)	**8.593 (.003)**	**3.999 (.046)**	0.772 (0.380)	0.003 (0.957)	1.768 (0.184)	1.024 (0.312)	**7.541 (0.006)**	3.767 (0.052)
Q6	0.391 (.532)	4.620 (.032)	1.004 (.316)	3.228 (0.072)	0.341 (0.559)	0.785 (0.376)	0.008 (0.928)	1.153 (0.283)	0.525 (0.469)
Q7	0.728 (.394)	0.101 (.751)	1.258 (.262)	0.539(0.463)	0.370 (0.543)	2.058(0.151)	0.026 (0.872)	**4.731 (0.030)**	0.077 (0.782)
Q8	2.855 (.091)	0.754 (.385)	2.273 (.132)	0.059 (.807)	0.861 (0.353)	1.573 (0.210)	3.329(0.068)	**15.181 (<0.001)**	2.972 (0.085)
Q9	**6.467(.011)**	**13.445 (<.001)**	**5.039 (.025)**	**50.405 (<.001)**	0.006 (0.939)	3.070 (0.080)	1.412 (0.235)	2.081 (0.149)	3.464 (0.063)
Q10	**5.230 (.022)**	**12.075 (.001)**	**9.731 (.002)**	**9.065 (.003)**	0.279 (0.579)	3.652 (0.056)	0.048 (0.827)	2.040 (0.153)	2.686 (0.101)
Q11	3.343 (.067)	0.792 (.373)	**7.620 (.006)**	0.869(.351)	0.109 (0.741)	**5.475 (0.019)**	1.881 (0.170)	1.045 (0.307)	0.812 (0.367)
Q12	**3.922 (.048)**	3.109 (.078)	**12.871 (<.001)**	3.014 (.083)	0.102 (0.750)	3.453 (0.063)	3.382 (0.066)	0.969 (0.325)	4.871 (0.027)
Q13	1.812 (.178)	1.037 (.308)	0.840 (.359)	5.875 (.015)	0.130 (0.719)	1.070 (0.301)	0.328 (0.567)	3.241 (0.072)	1.330 (0.249)
Q14	0.581 (.446)	0.001 (.990)	1.162(.281)	0.022 (.881)	0.433 (0.511)	0.004(0.949)	0.144 (0.705)	0.162 (0.688)	0.827 (0.363)

Q15	0.179 (.672)	0.134 (.714)	1.220 (.269)	2.341 (.126)	0.069 (0.793)	0.190 (.663)	0.976 (.323)	0.116 (0.733)	0.163 (0.687)
Q16	0.011 (.917)	**12.422 (<.001)**	**6.198 (.013)**	0.202 (.653)	2.621 (0.105)	0.041 (0.839)	0.020 (0.888)	0.060 (0.807)	0.039 (0.844)
Q17	2.530 (.112)	0.196 (.658)	0.001 (.994)	0.189 (.664)	1.338 (0.247)	**4.102 (0.043)**	2.233 (0.135)	0.326 (0.568)	**4.260 (0.039)**
Q18	0.417 (.519)	0.281 (.596)	2.614 (.106)	1.581 (.209)	**4.082 (0.043)**	0.325 0(.569)	1.002 (0.317)	0.403 (0.526)	**5.365 (0.021)**
Q19	0.031 (.860)	0.445 (.505)	0.201 (.654)	1.098 (.295)	0.707 (0.401)	0.384 (0.535)	1.158 (0.282)	2.257 (0.133)	0.345 (0.557)
Q20	0.263 (.608)	0.020 (.889)	0.243 (.622)	0.548 (.459)	0.697(0.404)	0.043 (0.836)	2.178 (0.140)	1.658 (0.198)	0.004 (0.947)
Q21	0.053 (.817)	0.120 (.729)	1.272 (.259)	2.394 (.122)	0.910 (0.340)	0.001 (0.997)	0.372 (0.542)	0.095 (0.758)	1.055 (0.304)
Q22	2.080 (.149)	**6.328 (.012)**	2.024 (.155)	3.164 (.075)	0.009 (0.923)	0.333 (0.564)	0.436 (0.509)	1.434 (0.231)	2.915 (0.088)
Q23	**4.412 (.036)**	1.057 (.304)	0.078 (.779)	0.141 (.707)	1.181 (0.277)	**5.245 (0.022)**	0.241 (0.623)	0.119 (0.730)	**5.483 (0.019)**
Q24	1.486 (.223)	**6.249 (.012)**	**3.940 (.047)**	0.157 (.692)	3.349 (0.067)	0.154 (0.695)	0.022 (0.883)	**5.545 (0.019)**	0.733 (0.392)

For this analysis the 2-point coding of questions 1-24 was used (as in the supplementary tables S1-S6). Chi-square values are presented followed by *p*-values in parenthesis; p-values lower than 0.05 are shown in bold.
